# Supplementary material for: Genome-Wide Screening in Human Embryonic Stem Cells Highlights the Hippo Signaling Pathway as Granting Synthetic Viability in ATM Deficiency
Source: Cells. 2023 May 29;12(11):1503. doi: 10.3390/cells12111503 (PMC10253227; doi:10.3390/cells12111503)
Supplement: Supplementary file 1 [file cells-12-01503-s001.zip › cells-2395729-supplementary.pdf]

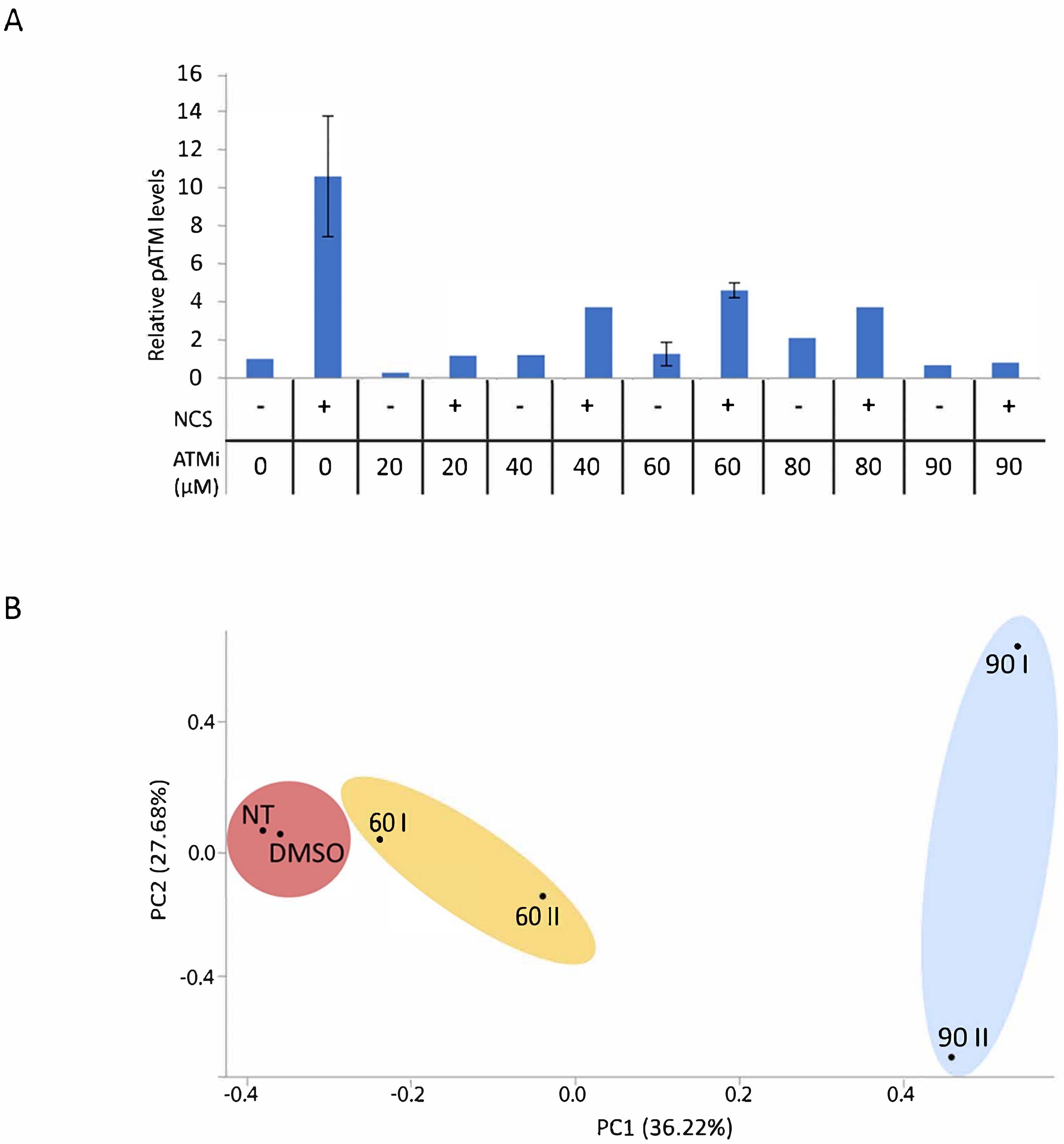

Supplementary Figure S1. Effect of ATMi on hESCs. **(A)** Bar graph summarizing western-blot analyses of phospho-ATM levels following treatment with increasing concentrations of ATMi, with or without DNA damage induction by NCS. Results are normalized to GAPDH protein levels, and to no treatment control. **(B)** PCA plot depicting the distance between count values of cells treated with different concentrations of ATMi.

A

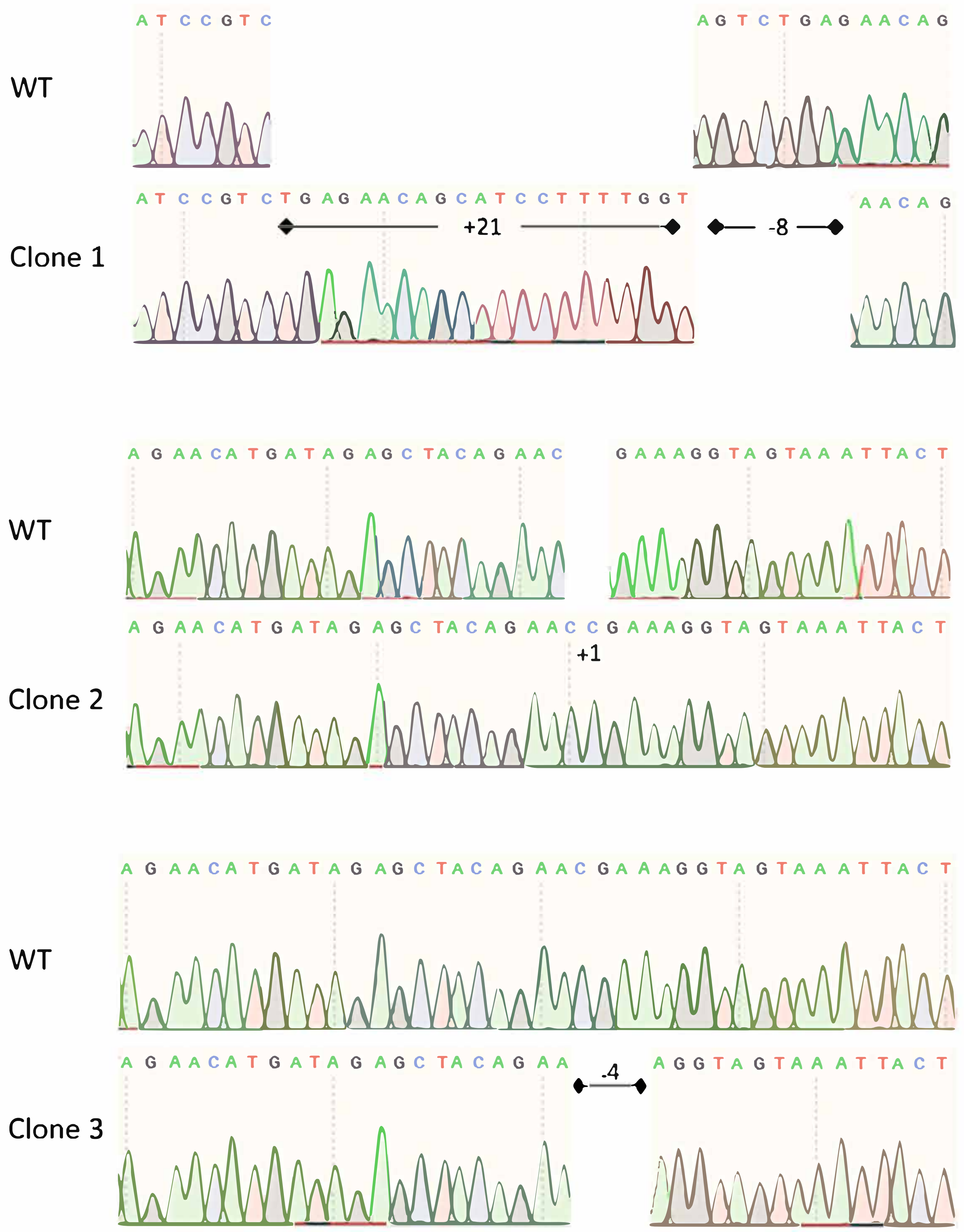

Supplementary Figure S2. Validation of ATM-knockout clones. **(A)** Sanger sequencing of ATM-knockout clones compared to WT. Relevant mutations are shown. Clones 1 and 2 had two mutations. In clone 1 the represented mutation resulted in frame shift, and in clone 2 the represented mutation gave rise to an early stop-codon shortly after mutation site. NT- no treatment.

Supplementary Table S1: Key resources and reagents

REAGENT or RESOURCE	SOURCE
**Antibodies**	
Mouse anti-human GPDH	abcam, ab8245
Rabbit anti-human ATM [Y170]	abcam, ab32420
Rabbit anti-human ATM (phospho S1981) [EP1890Y]	abcam, ab81292
Mouse anti-human Tubulin	abcam, ab56676
**Chemicals, Peptides, and Recombinant Proteins**	
mTeSR1 medium	STEMCELL Technologies
TrypLE Select	Thermo Fisher Scientific
Dulbecco's Modified Eagle's Medium	Sigma
Knockout Dulbecco's Modified Eagle's Medium (Knockout DMEM)	Thermo Fisher Scientific
Knockout Serum Replacement (KSR)	Thermo Fisher Scientific
l-Glutamine	Biological Industries
Nonessential amino acids	Biological Industries
Penicillin and streptomycin	Biological Industries
β-mercaptoethanol	Sigma
Human basic fibroblast growth factor (bFGF)	PeproTech
Gelatin	MP Biomedicals
Fetal bovine serum (FBS)	Thermo Fisher Scientific
polyethylenimine "Max" (PEI-Max)	Polysciences
Trypsin–EDTA	Biological Industries
Polybrene	Sigma
Puromycin dihydrochloride	Sigma
Neomycin (G418)	Sigma
Matrigel Growth Factor Reduced (GFR)	Corning
SB431542	Biogems
LDN193189	Biogems
N-2 supplement	Thermo Fisher
Ascorbic acid	Sigma
D-Glucose	BDH
16% PFA	Electron Microscopy Sciences
DPBS	Sigma-Aldrich
Bovine serum albumin (BSA)	MP Biomedicals
SDS	Bio-Rad
TRIS base	Bio-Lab
Glycine	J-T Baker
Methanol	Sigma
Bisacrylamide	Biological Industries
Tetramethylethylenediamine (TEMED)	Sigma
Nonfat milk powder	BD
Nitrocellulose blotting membrane	BioTrace
Hoechst33342	Sigma
KU-55933	Sigma

CGP3466B maleate	Tocris
Lats-IN-1	MedChemExpress (MCE)
Neocarzinostatin- NCS	Sigma
**Critical Commercial Assays**	
Enhanced Chemiluminescence Detection (ECL) kit	BioGate
Blood & Cell Culture DNA Midi Kit	Qiagen
Blood/Cell DNA Mini Kit	Geneaid
Nextera XT Index Kit, 96 indices, 384 samples	Illumina
RNeasy Mini Kit	QIAGEN
TruSeq RNA Library Prep Kit	Illumuna
**Deposited Data**	
RNaseq analysis of ATM-knockout hESCs with or without Hippo pathway inhibition	This study, ArrayExpress Accession number: E-MTAB-12600
**Experimental Models: Cell Lines**	
Haploid hESCs – h-pES10-based mutant library	Yilmaz et al., 2018
HEK293T	Laboratory of Robert Weinberg
**Oligonucleotides**	
sgRNAs for mutant generation, see Supplementary Table 2	This study
Primers for Library DNA amplification:	Yilmaz et al., 2018
5'-TCGTCGGCAGCGTCAGATGTGTATAAGAGACAG	
NNNNNNNNNNGGCTTTATATATCTTGTGGAAAGGACG-3' (forward).	
5'-GTCTCGTGGGCTCGGAGATGTGTATAAGAG	
ACAGACGGACTAGCCTTATTTTAACTTGC-3' (reverse).	
**Recombinant DNA**	
lentiCRISPR v2	A gift from Feng Zhang, Addgene Cat# 52961
lentiCRISPR v2-neo	A gift from Brett Stringer, Addgene Cat# 98292
pCMV-VSV-G	A gift from Bob Weinberg, Addgene Cat# 8454
psPAX2	A gift from Didier Trono, Addgene Cat# 12260
**Software and Algorithms**	
Flowjo software (FlowJo LLC)	BD
ImageJ software	NIH
Bowtie 2 aligner	Langmead and Salzberg, 2012
R (version 4.0.2)	R Core Team
STAR (version 2.5)	Dobin et al., 2013
EdgeR	Robinson et al., 2010
GSEA	Subramanian et al., 2005; Mootha et al., 2003
Illustrator	Adobe Ink.
OMIM	JHUSOM
STRING	Szklarczyk et al., 2019

**Others**	
BD Biosciences FACSAria III	BD Biosciences
Illumina NextSeq 500	Illumina
Gel Imager – ChemiDoc	Bio-Rad

Supplementary Table S2: Sequences of sgRNAs and primers used in this work

Name	Sequence	SOURCE
SAV1_1	CACCGGAGCGAGAAGGACTTCCTCC	This study
	AAACGGAGGAAGTCCTTCTCGCTCC	
SAV1_2	CACCGGAAGATTTACCCCTTCCTCC	This study
	AAACGGAGGAAGGGGTAAATCTTCC	
BAG6_1	CACCGGGCATTCCGGTCATGAACAG	This study
	AAACCTGTTCATGACCGGAATGCCC	
BAG6_2	CACCGGATGACCCTCGGGTGGCTGG	This study
	AAACCCAGCCACCCGAGGGTCATCC	
NF2_1	CACCGCGTCACCATGGACGCCGAGA	This study
	AAACTCTCGGCGTCCATGGTGACGC	
NF2_2	CACCGTCTTTGAGCCTACCTTGGCC	This study
	AAACGGCCAAGGTAGGCTCAAAGAC	
ATM_1	CACCGGGATGCTGTTCTCAGACTGA	Mancikova V, Peschelova H, Kozlova V, et al. *J Immunother Cancer*. 2020
	AAACTCAGTCTGAGAACAGCATCCC	
ATM_2	CACCGTGATAGAGCTACAGAACGAA	https://www.genscript.com/gRNA-detail/472/ATM-CRISPR-guide-RNA.html
	AAACTTCGTTCTGTAGCTCTATCAC	
ATM PCR Primer_1	AATGTTTCTGCGACCTGGCT	This study
	TGGTCAACAGAACATCAGCA	
ATM PCR Primer_2	GCCTTTGACCAGAATGTGCC	This study
	GGATCTCGAATCAGGCGCTT	

Supplementary Table S3: List of significantly enriched genes

Gene name	LogFC	P-value	FDR
TP53	7.512286008	0	0
NF2	6.108048769	3.44169E-15	1.54334E-11
PMAIP1	5.816833807	4.44089E-16	3.98281E-12
USP28	4.379876922	2.18714E-14	7.84614E-11
PTEN	4.317473273	1.33227E-15	7.96563E-12
BAX	3.585787711	6.89959E-12	2.06263E-08
BAG6	2.760837158	5.97437E-06	0.004584978
SAV1	2.602537902	1.24668E-07	0.000172013
TRIP12	2.550769023	0.000120099	0.039893013
TP53BP1	2.519246808	4.64766E-07	0.000521031
MNT	2.467479357	5.82482E-07	0.000614587
ZMYM2	2.463923901	1.01943E-11	2.61221E-08
SCAF8	2.414556554	2.52604E-05	0.012636094
RNF111	2.192468616	1.80591E-05	0.010122683
MAP4K4	2.101044185	7.94038E-10	1.58252E-06
TAOK1	2.071581426	5.40145E-05	0.022019509
SPRY4	2.041237627	4.07359E-05	0.01739715
RASA2	2.03722462	2.78411E-06	0.002496933
TADA2B	2.028638562	1.18275E-06	0.001116575
L3MBTL3	1.963170732	1.52174E-08	2.27462E-05
KDM5C	1.857713228	5.41038E-06	0.004411182
HIST1H1D	1.843353965	1.32638E-05	0.008496912
FAM193A	1.826806566	1.8864E-07	0.000225575
FLCN	1.758860772	1.62459E-07	0.000208145
PLEKHA1	1.743746907	3.65482E-09	5.95969E-06
CCDC6	1.740523005	1.1368E-06	0.001116575
CHEK2	1.733310331	4.62777E-10	1.0376E-06
CHI3L1	1.631426245	0.000171789	0.049035386
HYAL1	1.623068074	2.41799E-05	0.012636094
ACSL3	1.616442674	0.000136934	0.042347982
PGAP2	1.597455709	0.000123089	0.040142747
HIST1H1B	1.441349734	6.13478E-06	0.004584978
ZNF460	1.38140037	3.03954E-05	0.014735179
DCUN1D3	1.371981783	0.000112015	0.038638836
LZTR1	1.344562764	4.19006E-05	0.017478382
ZNF436	1.340002352	0.00015145	0.046043394
FBXO42	1.33165945	1.67989E-05	0.010007807
TSC2	1.276067528	1.00167E-05	0.006654428
RASA1	1.199555698	2.42699E-05	0.012636094
PAWR	1.023678405	2.5361E-05	0.012636094
MYL6	0.982927044	9.01526E-06	0.006219488
SLC26A10	0.978969533	3.77537E-05	0.016516769
METTL23	0.966129085	6.38672E-05	0.02545747
OSTN	0.930079719	0.00010315	0.037003887
PLD5	0.915255715	0.000131894	0.041810837
KRT27	0.880652745	8.27186E-05	0.030280056
SEC11C	0.861761275	0.000132866	0.041810837
HPCA	0.857193322	6.61946E-05	0.025811574

NEURL1B	0.746428307	3.57995E-05	0.016516769
